# Transient portal venous gas secondary to acute gastric dilatation

**DOI:** 10.1093/jscr/rjad705

**Published:** 2023-12-30

**Authors:** Shern Wai Koh, Michael F Bath, Manoj Nair

**Affiliations:** Department of General Surgery, North Middlesex University Hospital, Sterling Way, London, N18 1QX, United Kingdom; Department of General Surgery, North Middlesex University Hospital, Sterling Way, London, N18 1QX, United Kingdom; International Health Systems group, Department of Engineering, University of Cambridge, Cambridge, CB2 1PZ, United Kingdom; Department of General Surgery, North Middlesex University Hospital, Sterling Way, London, N18 1QX, United Kingdom

**Keywords:** general surgery, gastroenterology, radiology, ischaemia

## Abstract

A female patient in her 60s with a history of Parkinson’s disease developed epigastric and retrosternal chest pain, with associated vomiting. On examination, she had a distended abdomen with no focal peritonism. A computed tomography (CT) pulmonary angiogram was organized, which demonstrated no evidence of pulmonary emboli, but an incidental finding of gas within the liver peripheries and gastric fundal wall. A plain film abdominal radiograph demonstrated a significantly distended stomach. Thus, acute gastric dilatation was diagnosed. A nasogastric tube was introduced and intravenous fluids were given promptly. An urgent CT scan of abdomen and pelvis with intravenous contrast demonstrated interval reduction with only minor residual gas evident within the left lobe of the liver and gastric fundal wall. We report the case of transient portal venous gas, secondary to acute gastric dilatation, most likely caused by a combination of opioid analgesia and gastric dysmotility from Parkinson’s disease.

## Introduction

Acute gastric dilatation is one of the causes of portal venous gas, whereby a loss of normal protective mechanisms of the gastric mucosa due to a dilatation and ischaemia leads to gas entering the portal venous system via the stomach wall. Indeed, previous work has demonstrated that the vast majority of gastric pneumatosis results in portal venous gas [1]. Portal venous gas is often viewed as an ominous sign; however, we report the case of transient portal venous gas, secondary to acute gastric dilatation, with the patient making a full recovery following prompt treatment.

## Case report

A female patient in her 60s was admitted to hospital under the care of the orthopaedic team for an anterior shoulder joint dislocation following a mechanical fall. She underwent a manipulation under anaesthesia of her shoulder on Day 2 of admission and was provisionally planned for a subsequent joint replacement whilst an inpatient, being started on regular opioid analgesia in the interim. She had a past medical history of Parkinson’s disease, for which she took Co-Careldopa, and had undergone a laparoscopic anterior resection for a sigmoid cancer several years prior.

**Figure 1:**
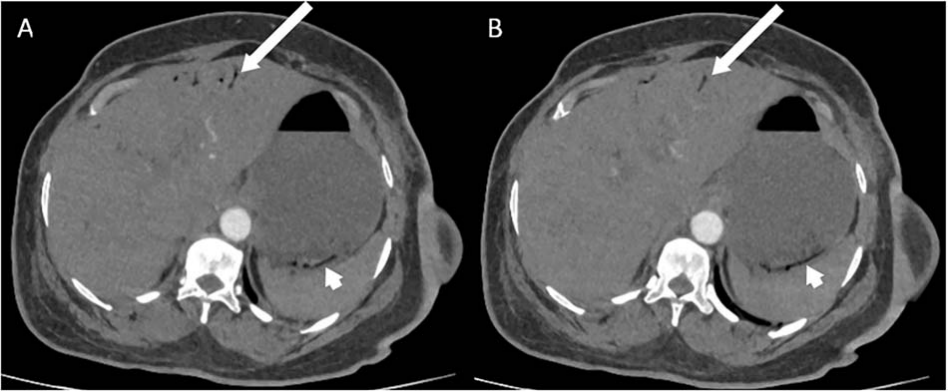
CT pulmonary angiogram images demonstrating moderate amount of branching linear gas in the liver (long arrows), most pronounced in (A) at the level of T11/12; gas within the wall of the gastric fundus (short arrows) can be appreciated more in (B) at the level of T11.

**Figure 2:**
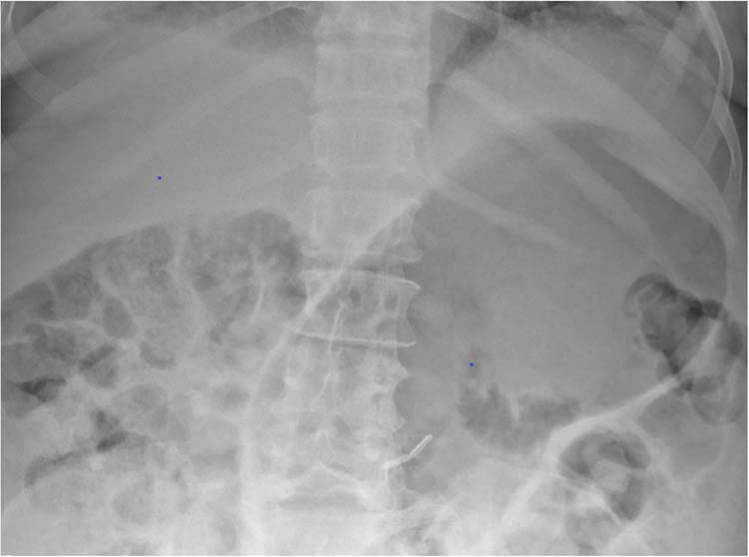
Plain film radiograph of the abdomen demonstrating a distended and gas-filled stomach.

Whilst awaiting her planned orthopaedic surgery, the patient developed epigastric and retrosternal chest pain on Day 13 of admission, associated with persistent vomiting and abdominal distension. On examination, she had a moderately distended abdomen, with no focal peritonism present. Initial blood tests performed showed mildly elevated inflammatory markers, normal serum troponin levels, and a raised D-dimer. As such, a computed tomography (CT) pulmonary angiogram was initially performed; whilst no evidence of pulmonary embolism was identified, an incidental finding of gas within the liver peripheries (Fig. 1A) and the gastric fundal wall was noted (Fig. 1B). A subsequent plain film abdominal radiograph performed showed a significantly distended stomach (Fig. 2).

**Figure 3:**
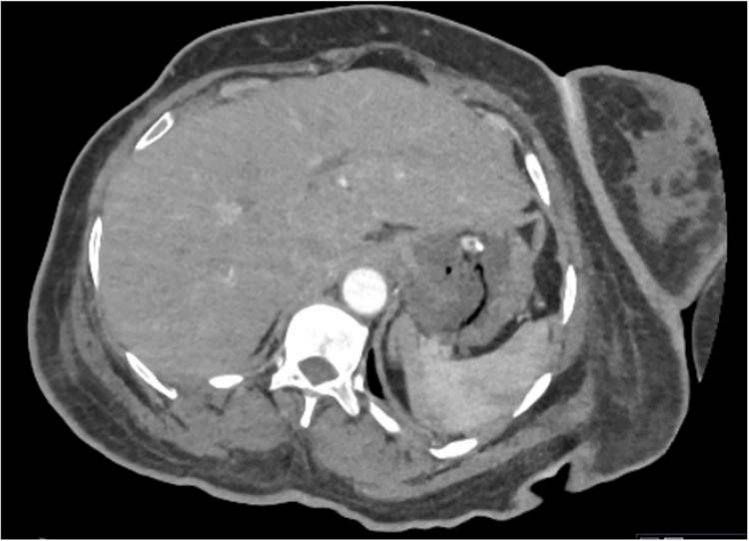
CT abdomen and pelvis image demonstrating interval resolution of portal venous gas, at the level of T11/12.

Based on the clinical and radiological picture, acute gastric dilatation was diagnosed, presumed secondary to a combination of opioid analgesia and gastric dysmotility from Parkinson’s disease. A nasogastric tube was introduced, intravenous fluids were given, and a venous blood gas was performed, which showed pH of 7.46 and lactate 1.0 mmol/L. An urgent CT scan of the abdomen and pelvis with intravenous contrast was performed; this demonstrated there had been interval reduction of the portal venous gas (Fig. 3), with only minor residual gas seen at the periphery of the left lobe of the liver and the gastric fundal wall. No other radiological features of visceral ischaemia within the abdomen were observed, with the coeliac axis and the collaterals from the phrenic arteries and superior mesenteric artery all patent.

The patient underwent an oesophago-gastro-duodenoscopy subsequently for direct intraluminal visualization of the stomach, which demonstrated gastritis and oesophagitis only, with no endoscopic evidence of ischaemia; histology results were consistent with reactive gastritis and Campylobacter-like organism (CLO) test was negative. She was started on high-dose proton pump inhibitor therapy and went on to make a full clinical recovery, starting back on normal diet on Day 17 of admission. She underwent an elective shoulder joint replacement 3 weeks after discharge.

## Discussion

We report the case of transient portal venous gas, secondary to acute gastric dilatation, most likely caused by a combination of opioid analgesia and gastric dysmotility from Parkinson’s disease. Portal venous gas due to gastric dilatation has previously been described [2], whereby a loss of normal protective mechanisms of the gastric mucosa from transient dilatation and ischaemia leads to gas entering the portal venous system via the stomach wall. However, this case demonstrates that prompt gastric decompression with effective placement of a nasogastric tube in this patient, with correction of the underlying causes, prevented further progression of the portal venous gas and allowed the patient to make a complete recovery.
